# The genome sequence of the Large Ear, *Amphipoea lucens* (Freyer, 1845)

**DOI:** 10.12688/wellcomeopenres.19287.1

**Published:** 2023-05-10

**Authors:** David Lees, Natural History Museum Genome Acquisition Lab, Darwin Tree of Life Barcoding collective, Wellcome Sanger Institute Tree of Life programme, Wellcome Sanger Institute Scientific Operations: DNA Pipelines collective, Tree of Life Core Informatics collective, Darwin Tree of Life Consortium

**Affiliations:** 1 Natural History Museum, London, England, UK

**Keywords:** Amphipoea lucens, Large Ear, genome sequence, chromosomal, Lepidoptera

## Abstract

We present a genome assembly from an individual male *Amphipoea lucens* (the Large Ear; Arthropoda; Insecta; Lepidoptera; Noctuidae). The genome sequence is 647.7 megabases in span. Most of the assembly is scaffolded into 31 chromosomal pseudomolecules, including the assembled Z sex chromosome. The mitochondrial genome has also been assembled and is 15.3 kilobases in length.

## Species taxonomy

Eukaryota; Metazoa; Ecdysozoa; Arthropoda; Hexapoda; Insecta; Pterygota; Neoptera; Endopterygota; Lepidoptera; Glossata; Ditrysia; Noctuoidea; Noctuidae; Noctuinae; Apameini; *Amphipoea*; *Amphipoea lucens* (Freyer, 1845) (NCBI:txid987875).

## Background

The Large Ear, *Amphipoea lucens*, is a medium-sized noctuid moth, often marginally larger than other species in the genus (forewing length up to 17 mm), but with similar markings such as a usually orange orbicular and reniform stigmata on the forewing. The moth is thus hard to separate on external features, despite strong bands on the underside wings, with the male genitalia with the longer arm on the clasper usually curved distally ( Waring *et al.*, 2017) and with genitalic differences in the female also ( British Lepidoptera). It is monovoltine, flying from late July to early October in the UK ( Randle *et al.*, 2019), and overwintering as an egg. 

The Large Ear is found in damp habitats, such as acid moorland and marshes, the larva feeding on purple moor grass ( *Molinia caerulea* (L.) Moench) and common cottongrass ( *Eriophorum angustifolium* Honck.); the adult can be found at flowers of rushes and heathers ( Waring *et al.*, 2017).

*Amphipoea lucens* is generally widespread in the western Palaearctic, from southern Scandinavia to Italy; there seem to be relatively few records for western Russia and it is found as far East as northern Japan and Korea ( GBIF Secretariat, 2022). Populations in the UK appear to have declined since 1970 ( Conrad *et al.*, 2006; Randle *et al.*, 2019). In the UK it is local and more prevalent towards the north as far as the Shetlands ( NBN Atlas Partnership, 2021) where records have been considered to represent immigrant individuals ( Waring *et al.*, 2017).

**Figure 1.**
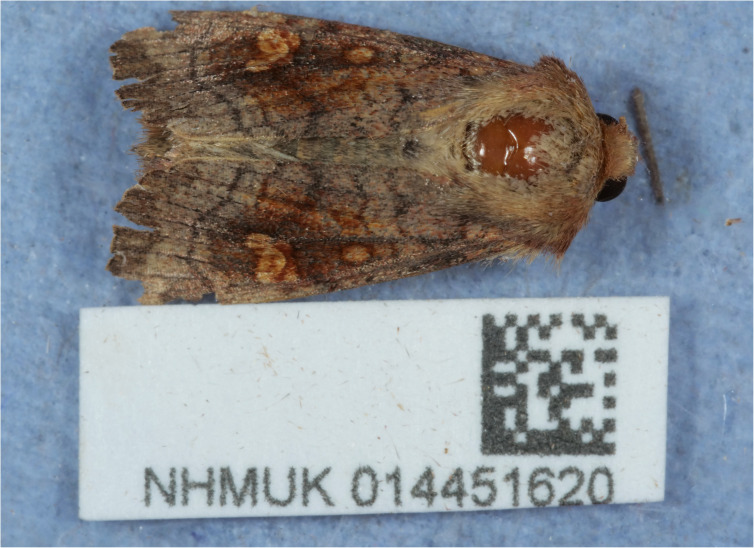
Photograph of the *Amphipoea lucens* (ilAmpLuce6) specimen used for genome sequencing.

The Large Ear is currently placed in the noctuid tribe Apameini and the genus was included in a distal part of this tribe in ( Toussaint *et al.*, 2012) ( Figure 1) as sister to a group containing the genera *Lateroligia*, *Coenobia*, *Luperina*, *Mesapamea* and *Mesoligia*. The genome sequence should not only be useful in phylogeny but in studies of speciation, considering also that the species of *Amphipoea* species are notoriously difficult to identify externally. The current picture is further clouded by mitochondrial data in that two DNA barcode clusters occur on BOLD (8 March 2023), both found in the UK. Some of exemplars fall in the cluster BOLD:AAB5368 (mostly identified as *Amphipoea fucosa* (Freyer, 1830), others as *A. lucens* and a few as *A. crinanensis* (Burrows, 1908)), whilst other exemplars fall in the BIN BOLD:AAC7752 (most identified as *A. oculea* (Linnaeus, 1761) and a few as *A. lucens*). BOLD:AAC7752 is about 2.37% pairwise divergent from BOLD:AAB5368.

The genome of *Amphipoea lucens* was sequenced as part of the Darwin Tree of Life Project, a collaborative effort to sequence all named eukaryotic species in the Atlantic Archipelago of Britain and Ireland. Here we present a chromosomally complete genome sequence for *Amphipoea lucens*, based on one male specimen from Beinn Eighe National Nature Reserve, Scotland.

## Genome sequence report

The genome was sequenced from one male *Amphipoea lucens* ( Figure 1) collected from Beinn Eighe (See Methods). A total of 36-fold coverage in Pacific Biosciences single-molecule HiFi long reads was generated. Primary assembly contigs were scaffolded with chromosome conformation Hi-C data. Manual assembly curation corrected 17 missing joins or mis-joins and removed nine haplotypic duplications, reducing the assembly length by 1.96% and the scaffold number by 10.42%.

**Figure 2.**
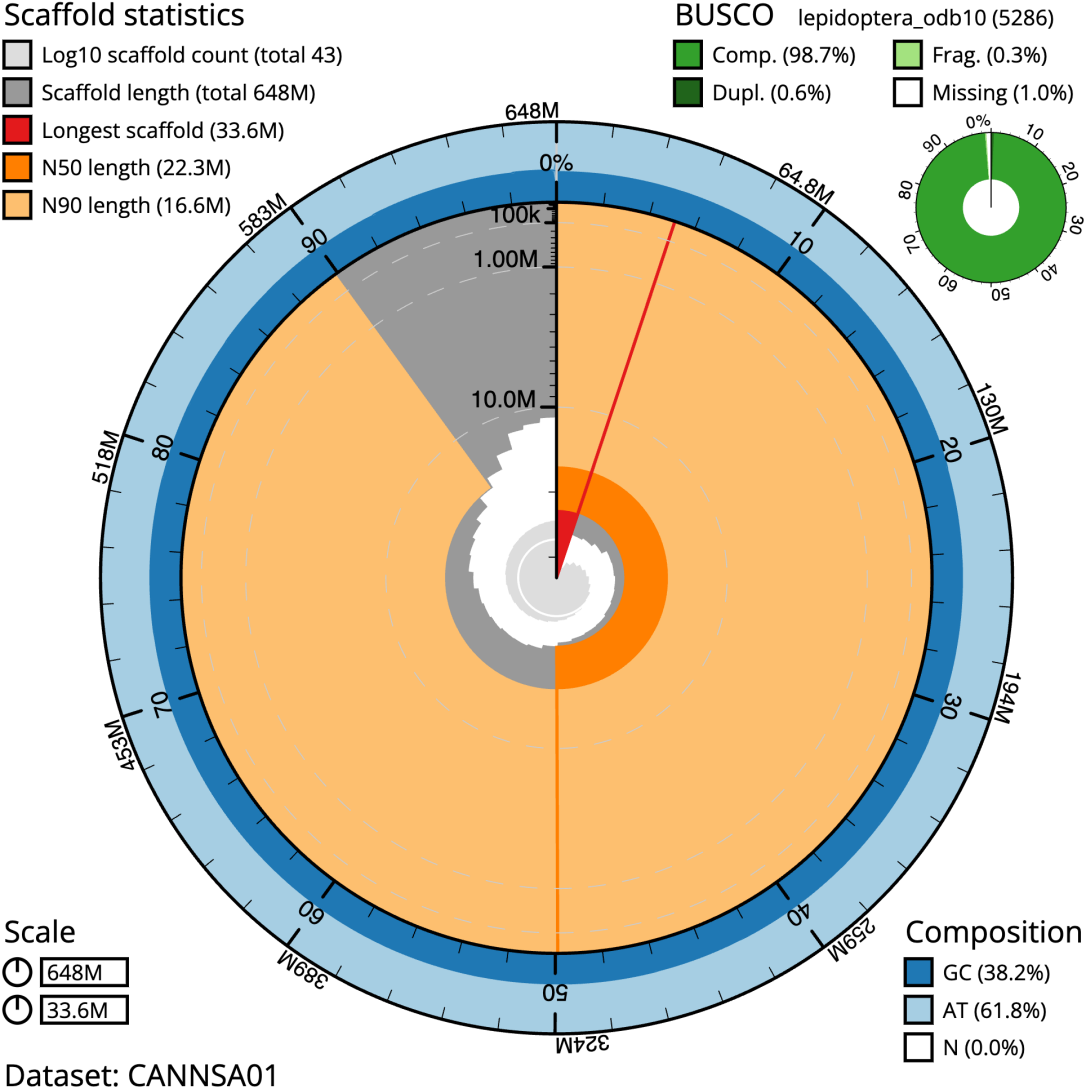
Genome assembly of *Amphipoea lucens*, ilAmpLuce6.1: metrics. The BlobToolKit Snailplot shows N50 metrics and BUSCO gene completeness. The main plot is divided into 1,000 size-ordered bins around the circumference with each bin representing 0.1% of the 647,718,368 bp assembly. The distribution of scaffold lengths is shown in dark grey with the plot radius scaled to the longest scaffold present in the assembly (33,593,231 bp, shown in red). Orange and pale-orange arcs show the N50 and N90 scaffold lengths (22,308,434 and 16,611,211 bp), respectively. The pale grey spiral shows the cumulative scaffold count on a log scale with white scale lines showing successive orders of magnitude. The blue and pale-blue area around the outside of the plot shows the distribution of GC, AT and N percentages in the same bins as the inner plot. A summary of complete, fragmented, duplicated and missing BUSCO genes in the lepidoptera_odb10 set is shown in the top right. An interactive version of this figure is available at https://blobtoolkit.genomehubs.org/view/ilAmpLuce6.1/dataset/CANNSA01/snail.

The final assembly has a total length of 647.7 Mb in 43 sequence scaffolds with a scaffold N50 of 22.3 Mb ( Table 1). Most (99.91%) of the assembly sequence was assigned to 31 chromosomal-level scaffolds, representing 30 autosomes, and the Z sex chromosome. Chromosome-scale scaffolds confirmed by the Hi-C data are named in order of size ( Figure 2– Figure 5; Table 2). While not fully phased, the assembly deposited is of one haplotype. Contigs corresponding to the second haplotype have also been deposited. The mitochondrial genome was also assembled and can be found as a contig within the multifasta file of the genome submission.

**Table 1.** Genome data for *Amphipoea lucens*, ilAmpLuce6.1.

Project accession data		
Assembly identifier	ilAmpLuce6.1	
Species	*Amphipoea lucens*	
Specimen	ilAmpLuce6	
NCBI taxonomy ID	987875	
BioProject	PRJEB55798	
BioSample ID	SAMEA14448313	
Isolate information	ilAmpLuce6, male, head and thorax (genome sequencing and Hi-C scaffolding)	
Assembly metrics *		*Benchmark*
Consensus quality (QV)	67.9	*≥ 50*
*k*-mer completeness	100%	*≥ 95%*
BUSCO **	C:98.7%[S:98.1%,D:0.6%], F:0.3%,M:1.0%,n:5,286	*C ≥ 95%*
Percentage of assembly mapped to chromosomes	99.91%	*≥ 95%*
Sex chromosomes	Z chromosome	*localised homologous pairs*
Organelles	Mitochondrial genome assembled	*complete single alleles*
Raw data accessions		
PacificBiosciences SEQUEL II	ERR10168736	
Hi-C Illumina	ERR10177753	
Genome assembly		
Assembly accession	GCA_947508005.1	
*Accession of alternate haplotype*	GCA_947507765.1	
Span (Mb)	647.7	
Number of contigs	109	
Contig N50 length (Mb)	11.3	
Number of scaffolds	43	
Scaffold N50 length (Mb)	22.3	
Longest scaffold (Mb)	33.6	

^*^Assembly metric benchmarks are adapted from column VGP-2020 of “Table 1: Proposed standards and metrics for defining genome assembly quality” from ( Rhie *et al.*, 2021). ^**^ BUSCO scores based on the lepidoptera_odb10 BUSCO set using v5.3.2. C = complete [S = single copy, D = duplicated], F = fragmented, M = missing, n = number of orthologues in comparison. A full set of BUSCO scores is available at https://blobtoolkit.genomehubs.org/view/ilAmpLuce6.1/dataset/CANNSA01/busco.

**Figure 3.**
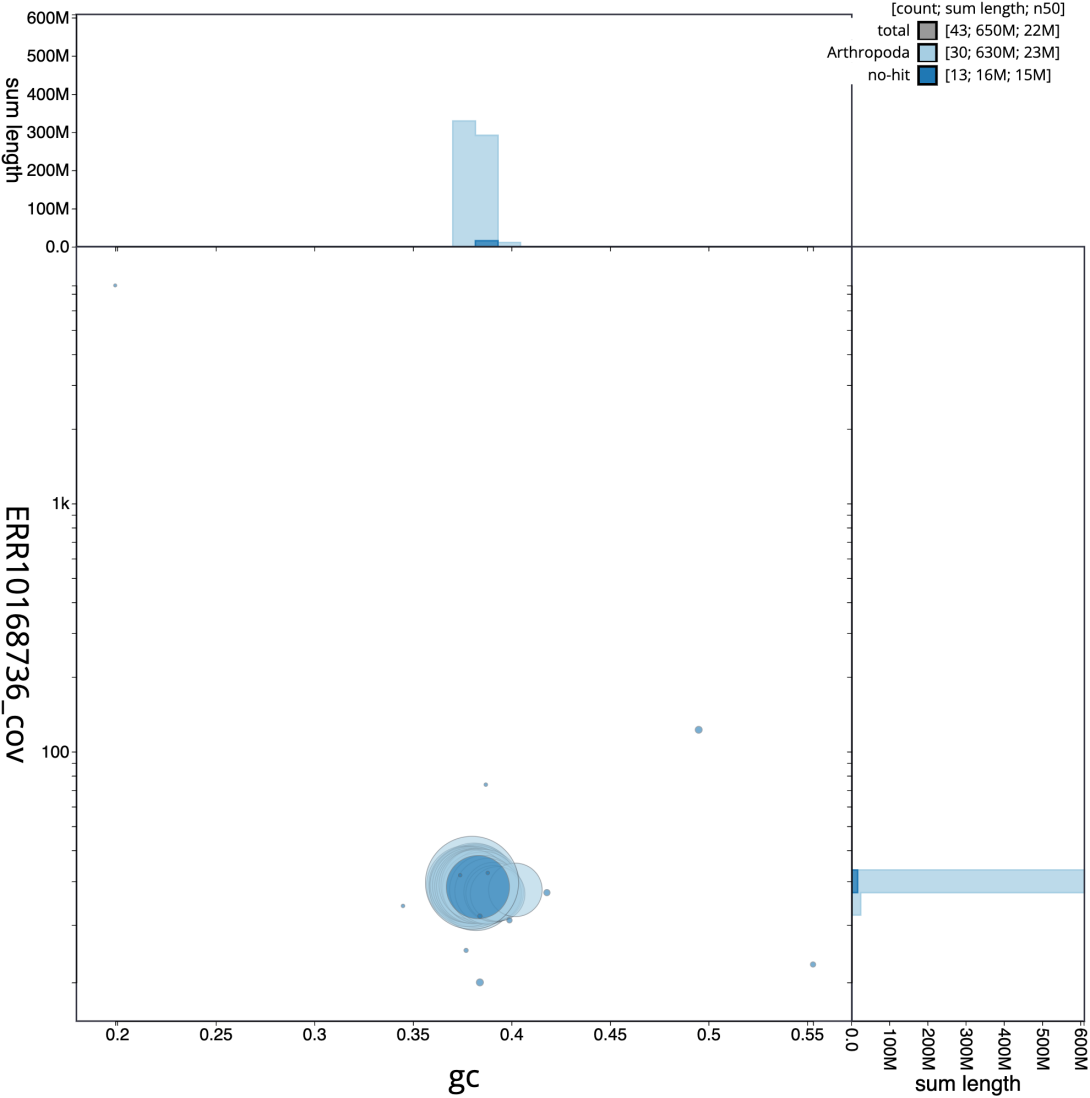
Genome assembly of *Amphipoea lucens*, ilAmpLuce6.1: GC coverage. BlobToolKit GC-coverage plot. Scaffolds are coloured by phylum. Circles are sized in proportion to scaffold length. Histograms show the distribution of scaffold length sum along each axis. An interactive version of this figure is available at https://blobtoolkit.genomehubs.org/view/ilAmpLuce6.1/dataset/CANNSA01/blob.

**Figure 4.**
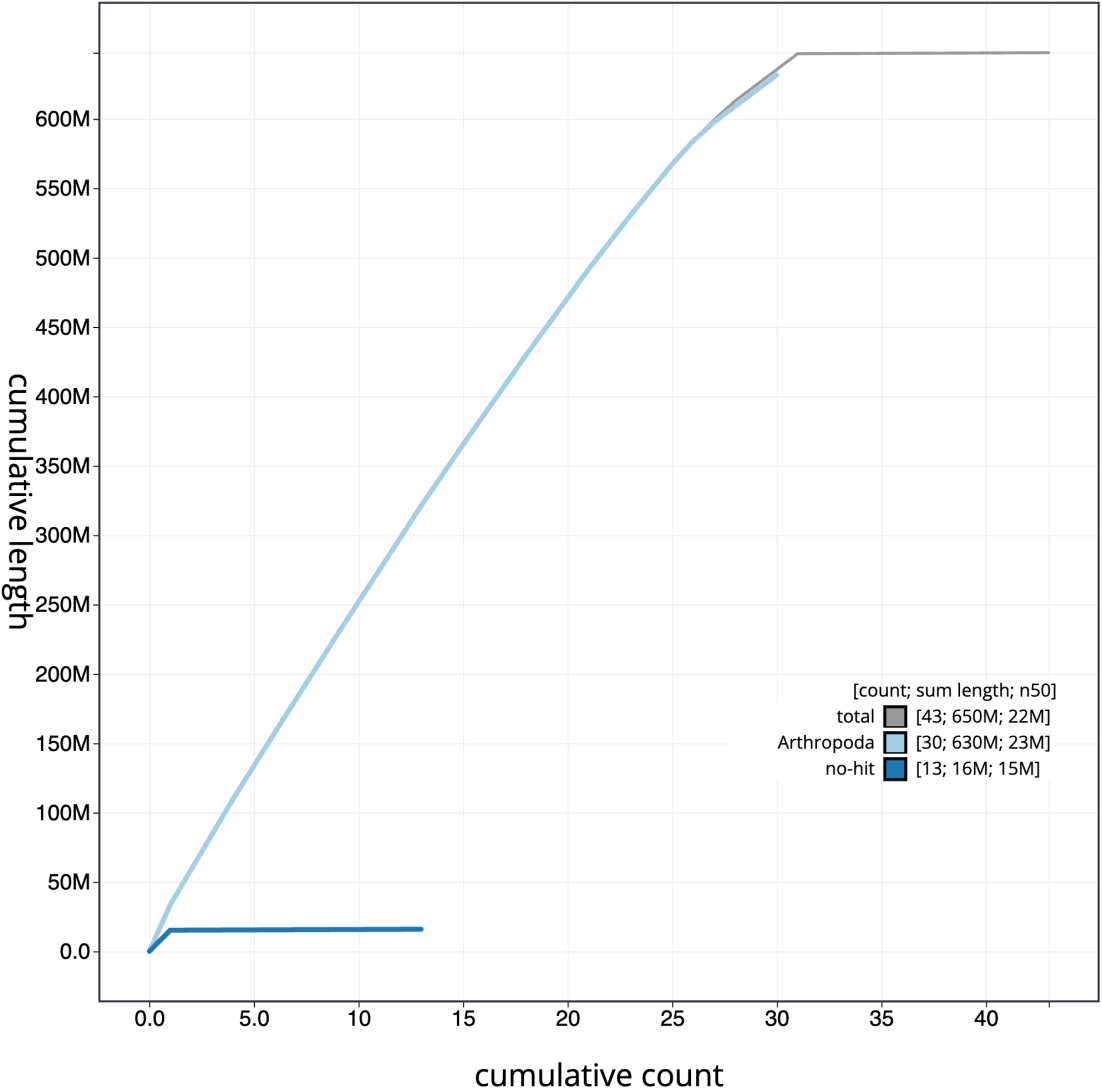
Genome assembly of *Amphipoea lucens*, ilAmpLuce6.1: cumulative sequence. BlobToolKit cumulative sequence plot. The grey line shows cumulative length for all scaffolds. Coloured lines show cumulative lengths of scaffolds assigned to each phylum using the buscogenes taxrule. An interactive version of this figure is available at https://blobtoolkit.genomehubs.org/view/ilAmpLuce6.1/dataset/CANNSA01/cumulative.

**Figure 5.**
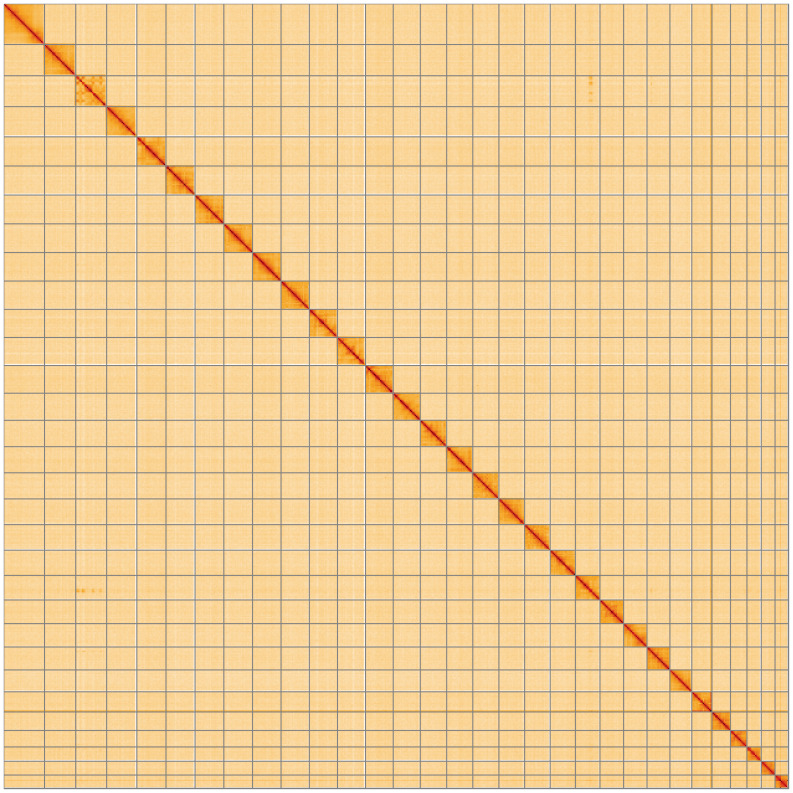
Genome assembly of *Amphipoea lucens*, ilAmpLuce6.1: Hi-C contact map. Hi-C contact map of the ilAmpLuce6.1 assembly, visualised using HiGlass. Chromosomes are shown in order of size from left to right and top to bottom. An interactive version of this figure may be viewed at https://genome-note-higlass.tol.sanger.ac.uk/l/?d=LlzrRwP5TQK2GbsjsUomLg.

**Table 2.** Chromosomal pseudomolecules in the genome assembly of *Amphipoea lucens*, ilAmpLuce6.

INSDC accession	Chromosome	Size (Mb)	GC%
OX382356.1	1	25.78	38.1
OX382357.1	2	25.48	38.2
OX382358.1	3	24.9	38.2
OX382359.1	4	24.04	38.3
OX382360.1	5	23.96	37.7
OX382361.1	6	23.76	37.8
OX382362.1	7	23.67	37.9
OX382363.1	8	23.53	38.2
OX382364.1	9	23.29	38
OX382365.1	10	23.24	38.1
OX382366.1	11	23.12	37.8
OX382367.1	12	22.86	38.1
OX382368.1	13	22.31	37.8
OX382369.1	14	21.78	37.9
OX382370.1	15	21.54	38
OX382371.1	16	21.5	38.1
OX382372.1	17	21.25	38.3
OX382373.1	18	21.06	38.3
OX382374.1	19	20.83	38.3
OX382375.1	20	20.21	38.6
OX382376.1	21	19.53	38.6
OX382377.1	22	19.28	38.3
OX382378.1	23	18.87	38.1
OX382379.1	24	18.07	38.6
OX382380.1	25	16.61	38.8
OX382381.1	26	15.28	38.3
OX382382.1	27	13.56	39.1
OX382383.1	28	11.8	39.1
OX382384.1	29	11.39	39.3
OX382385.1	30	10.93	40.2
OX382355.1	Z	33.59	38
OX382386.1	MT	0.02	20.1
-	unplaced	0.69	42.8

The estimated Quality Value (QV) of the final assembly is 67.9 with *k*-mer completeness of 100%, and the assembly has a BUSCO v5.3.2 completeness of 98.7% (single 98.1%, duplicated 0.6%) using the lepidoptera_odb10 reference set ( *n* = 5,286).

Metadata about the specimens, spectral estimates, sequencing runs, contaminants and pre-curation assembly statistics can be found here.

## Methods

### Sample acquisition and nucleic acid extraction

A male *Amphipoea lucens* specimen (ilAmpLuce6) was collected from Beinn Eighe National Nature Reserve, Scotland (latitude 57.63, longitude –5.35) on 10 September 2021, using an aerial net. The specimen was collected and identified by David Lees (Natural History Museum) and dry frozen at –80°C.

DNA was extracted at the Tree of Life laboratory, Wellcome Sanger Institute (WSI). The ilAmpLuce6 sample was weighed and dissected on dry ice with tissue set aside for Hi-C sequencing. Head and thorax tissue was disrupted using a Nippi Powermasher fitted with a BioMasher pestle. High molecular weight (HMW) DNA was extracted using the Qiagen MagAttract HMW DNA extraction kit. HMW DNA was sheared into an average fragment size of 12–20 kb in a Megaruptor 3 system with speed setting 30. Sheared DNA was purified by solid-phase reversible immobilisation using AMPure PB beads with a 1.8X ratio of beads to sample to remove the shorter fragments and concentrate the DNA sample. The concentration of the sheared and purified DNA was assessed using a Nanodrop spectrophotometer and Qubit Fluorometer and Qubit dsDNA High Sensitivity Assay kit. Fragment size distribution was evaluated by running the sample on the FemtoPulse system.

### Sequencing

Pacific Biosciences HiFi circular consensus DNA sequencing libraries were constructed according to the manufacturers’ instructions. DNA sequencing was performed by the Scientific Operations core at the WSI on the Pacific Biosciences SEQUEL II (HiFi) instrument. Hi-C data were also generated from tissue of ilAmpLuce6 using the Arima v2 kit and sequenced on the Illumina NovaSeq 6000 instrument.

### Genome assembly, curation and evaluation

Assembly was carried out with Hifiasm ( Cheng *et al.*, 2021) and haplotypic duplication was identified and removed with purge_dups ( Guan *et al.*, 2020). The assembly was then scaffolded with Hi-C data ( Rao *et al.*, 2014) using YaHS ( Zhou *et al.*, 2023). The assembly was checked for contamination as described previously ( Howe *et al.*, 2021). Manual curation was performed using HiGlass ( Kerpedjiev *et al.*, 2018) and Pretext ( Harry, 2022). The mitochondrial genome was assembled using MitoHiFi ( Uliano-Silva *et al.*, 2022), which runs MitoFinder ( Allio *et al.*, 2020) or MITOS ( Bernt *et al.*, 2013), and uses these annotations to select the final mitochondrial contig and to ensure the general quality of the sequence. To evaluate the assembly, MerquryFK was used to estimate consensus quality (QV) scores and *k*-mer completeness ( Rhie *et al.*, 2020). The genome was analysed within the BlobToolKit environment ( Challis *et al.*, 2020) and BUSCO scores ( Manni *et al.*, 2021; Simão *et al.*, 2015) were calculated. Table 3 contains a list of software tool versions and sources.

**Table 3.** Software tools and versions used.

Software tool	Version	Source
BlobToolKit	4.0.7	Challis *et al.*, 2020
Hifiasm	0.16.1-r375	Cheng *et al.*, 2021
HiGlass	1.11.6	Kerpedjiev *et al.*, 2018
MitoHiFi	2	Uliano-Silva *et al.*, 2022
PretextView	0.2	Harry, 2022
purge_dups	1.2.3	Guan *et al.*, 2020
YaHS	yahs-1.1.91eebc2	Zhou *et al.*, 2023

### Ethics and compliance issues

The materials that have contributed to this genome note have been supplied by a Darwin Tree of Life Partner. The submission of materials by a Darwin Tree of Life Partner is subject to the Darwin Tree of Life Project Sampling Code of Practice. By agreeing with and signing up to the Sampling Code of Practice, the Darwin Tree of Life Partner agrees they will meet the legal and ethical requirements and standards set out within this document in respect of all samples acquired for, and supplied to, the Darwin Tree of Life Project. All efforts are undertaken to minimise the suffering of animals used for sequencing. Each transfer of samples is further undertaken according to a Research Collaboration Agreement or Material Transfer Agreement entered into by the Darwin Tree of Life Partner, Genome Research Limited (operating as the Wellcome Sanger Institute), and in some circumstances other Darwin Tree of Life collaborators.

## Data Availability

European Nucleotide Archive: *Amphipoea lucens.* Accession number PRJEB55798; https://identifiers.org/ena.embl/PRJEB55798 ( Wellcome Sanger Institute, 2022) The genome sequence is released openly for reuse. The *Amphipoea lucens* genome sequencing initiative is part of the Darwin Tree of Life (DToL) project. All raw sequence data and the assembly have been deposited in INSDC databases. The genome will be annotated using available RNA-Seq data and presented through the Ensembl pipeline at the European Bioinformatics Institute. Raw data and assembly accession identifiers are reported in Table 1.

## Author information

Members of the Natural History Museum Genome Acquisition Lab are listed here: https://doi.org/10.5281/zenodo.4790042.

Members of the Darwin Tree of Life Barcoding collective are listed here: https://doi.org/10.5281/zenodo.4893703.

Members of the Wellcome Sanger Institute Tree of Life programme are listed here: https://doi.org/10.5281/zenodo.4783585.

Members of Wellcome Sanger Institute Scientific Operations: DNA Pipelines collective are listed here: https://doi.org/10.5281/zenodo.4790455.

Members of the Tree of Life Core Informatics collective are listed here: https://doi.org/10.5281/zenodo.5013541.

Members of the Darwin Tree of Life Consortium are listed here: https://doi.org/10.5281/zenodo.4783558.
