# Supplementary material for: The genome sequence of the Large Ear, Amphipoea lucens (Freyer, 1845)
Source: Wellcome Open Res. 2023 May 10;8:204. [Version 1] doi: 10.12688/wellcomeopenres.19287.1 (PMC10352624; doi:10.12688/wellcomeopenres.19287.1)
Supplement: Peer review history

# Peer review: Reviewer response for version 1

**DOI:** 10.21956/wellcomeopenres.21370.r57634

**Published:** 2023-08-14

**Authors:** Tarekegn Fite Duressa

**Affiliations:** 1 School of Plant Sciences, College of Agriculture and Environmental Sciences, Haramaya University, Dire Dawa, Dire Dawa, Ethiopia; 2 International Centre of Insect Physiology and Ecology (Icipe), Addis Ababa, Ethiopia

The genome assembly paper for the Large Ear, Amphipoea lucens has exquisite writing and discusses the sequencing and assembly procedures in depth. The following are some minor comments and suggestions that authors may or may not accept:

- Protein-coding genes have to be indicated-Using Large Ear, or/and *Amphipoea* *lucens* is not a good way of scientific naming and using them.
- Use either a common name or a scientific name throughout.
- Once *Amphipoea* *lucens* is used, except when it is used as the first word of new sentences, you can use *A. lucens* throughout the paper.

Are sufficient details of methods and materials provided to allow replication by others?

Yes

Is the rationale for creating the dataset(s) clearly described?

Yes

Are the datasets clearly presented in a useable and accessible format?

Yes

Are the protocols appropriate and is the work technically sound?

Yes

Reviewer Expertise:

Genetic diversity, phylogenetics, ecology, biodiversity, biological control, population structure, IPM

I confirm that I have read this submission and believe that I have an appropriate level of expertise to confirm that it is of an acceptable scientific standard, however I have significant reservations, as outlined above.

# Peer review: Reviewer response for version 1

**DOI:** 10.21956/wellcomeopenres.21370.r57642

**Published:** 2023-07-17

**Authors:** Morthala Shankara Sai Reddy

**Affiliations:** 1 Department of Entomology, Dr Rajendra Prasad Central Agricultural University, Samastipur, Bihar, India

The report presents impressive work for the field of insect genomics with the sequencing and assembly of the genome of *Amphipoea lucens*, a captivating medium-sized noctuid moth. The meticulous effort involved in collecting a male specimen from Scotland and employing advanced techniques like Pacific Biosciences long reads and chromosome conformation Hi-C data has resulted in a remarkable accomplishment. The comprehensive assembly, composed of 43 sequence scaffolds spanning 647.7 Mb, showcases the dedication and expertise of the researchers. The assignment of 31 chromosomal-level scaffolds, including the identification of autosomes and the Z sex chromosome, demonstrates the meticulousness of the analysis. The assembly quality, highlighted by a high QV score of 67.9 and a remarkable BUSCO completeness of 98.7%, attests to the precision and accuracy of the work. Furthermore, the successful assembly of the mitochondrial genome adds to the significance of this endeavor. This invaluable genome sequence promises to greatly contribute to our understanding of the phylogenetics and speciation processes within the fascinating genus Amphipoea. Overall, this study represents a significant leap forward in genomic research, shedding light on the intricate molecular landscape of this captivating moth species.

Are sufficient details of methods and materials provided to allow replication by others?

Yes

Is the rationale for creating the dataset(s) clearly described?

Yes

Are the datasets clearly presented in a useable and accessible format?

Yes

Are the protocols appropriate and is the work technically sound?

Yes

Reviewer Expertise:

Molecular Entomology and Host Plant Resistance

I confirm that I have read this submission and believe that I have an appropriate level of expertise to confirm that it is of an acceptable scientific standard.

# Peer review: Reviewer response for version 1

**DOI:** 10.21956/wellcomeopenres.21370.r57639

**Published:** 2023-06-02

**Authors:** Amali Thrimawithana

**Affiliations:** 1 Plant and Food Research Institute of New Zealand Ltd, Auckland, New Zealand

The article was on the genome of large ear, a moth found across Europe. The method used for the assembly is good - latest potential workflows. Was great to see a great level of completion achieved and am looking forward to seeing the completion of annotation and comparative analysis thereafter. 

 Just couple of minor edit suggestions, I noticed large ear and scientific name is interchangeably used between paragraphs - it would be good to have consistency.

 version of MerquryFK is missing.

 It might be good to briefly mention the throughput, N50 etc of pacbio data generated and hi-c data.

 Again great genome report, looking forward to the next stage.

Are sufficient details of methods and materials provided to allow replication by others?

Yes

Is the rationale for creating the dataset(s) clearly described?

Yes

Are the datasets clearly presented in a useable and accessible format?

Yes

Are the protocols appropriate and is the work technically sound?

Yes

Reviewer Expertise:

bioinformatics, plant and animal genomics/transcriptomics, metagenomic

I confirm that I have read this submission and believe that I have an appropriate level of expertise to confirm that it is of an acceptable scientific standard.
